# Intersecting Pathologies: Vulvar Cancer Complicated by a Fusobacterium necrophorum Infection

**DOI:** 10.7759/cureus.73420

**Published:** 2024-11-11

**Authors:** Todd R Anderson, Emily Carletto, Valeria Barreto-Nadal, Emily Langston, Michael Jones

**Affiliations:** 1 Obstetrics and Gynecology, Campbell University School of Osteopathic Medicine, Lillington, USA; 2 Pediatrics, Campbell University School of Osteopathic Medicine, Fayetteville, USA; 3 Obstetrics and Gynecology, Campbell University School of Osteopathic Medicine, Fayetteville, USA; 4 Obstetrics and Gynecology, Cape Fear Valley Health, Fayetteville, USA

**Keywords:** cancer, case report, fusobacterium necrophorum, hpv, vulvar cancer, women’s health

## Abstract

Vulvar cancer represents a small minority of annual cancer cases in America. This malignancy affects more than the physical health of the patient. This malignancy also has a negative impact on women’s psychological and sexual health. This case pertains to a 49-year-old woman (G1P1001) who presented with right groin pain, fever, and chills for three days. She reported experiencing vaginal bleeding during the intercourse and had been able to palpate a mass inside her vagina for the past five years. A pelvic exam revealed a 6 cm tender mass on the left vaginal wall, which was friable and hemorrhagic on palpation, along with associated lymphadenopathy. The patient was taken to the operating room where the exophytic mass and lymphadenopathy were biopsied. Necrotic lymph nodes were removed and sent for pathology.

Pathology confirmed moderately differentiated squamous cell carcinoma of the vulva. The lymph node pathology report also indicated that the necrotic nodes were infected with *Fusobacterium necrophorum*. Given the extent of the tumor, it was deemed largely inoperable, and the patient was recommended local radiation therapy.

The patient was diagnosed with stage III vulvar cancer. This case highlights the intersection of gynecology-oncology and infectious disease, with a never-before-documented bacterial infection in the context of vulvar cancer. *Fusobacterium necrophorum* has been previously associated with colorectal cancers, liver abscesses, and ovarian abscesses. Further ingestion is needed to understand the role of this pathogen in the development of vulvar cancer.

## Introduction

Vulvar cancer represents a minority of annual cancer cases in America, representing only 0.7% of all cancers in women. It is estimated that in 2024, 6,900 cases of vulvar cancer will be diagnosed and 1,630 cases will result in death. This cancer is primarily diagnosed in elderly women with at average age of diagnosis of 68 years. Atypical squamous cells account for 90% of these cases in histological analysis [1,2]. The risk factors for this malignancy include human papillomavirus (HPV), increasing age, smoking, prior irritation, and immunodeficiency [3]. The gold standard for diagnosing this malignancy is histology. The treatment includes surgical excision, radiation, and chemotherapy directed by the healthcare team [4,5]. The lymph node histology is the most critical prognostic factor for survival, highlighting the importance of pelvic lymphadenectomy. Patients with metastatic lymph nodes have a 10-year survival of 65% compared to node-negative patients with a 10-year survival of 91% [6]. It is important to note that this malignancy not only affects a patient’s physical health, but it also can negatively impact the patient’s psychological and sexual health.

*Fusobacterium necrophorum* is a gram-negative anaerobic bacillus that has been cited in infections such as hepatic and oropharyngeal abscesses [7-9]. This pathogen is also cited in Lemierre’s disease, which is pharyngitis that spreads to lateral pharyngeal space, leading to septic thrombophlebitis of internal jugular veins [7,10]. Rarer presentations of this pathogen are found in association with colorectal cancer. Additionally, this pathogen has also been linked to infections in gynecological organs, including ovarian abscesses, however not cited in gynecological malignancies [11-13].

## Case presentation

A 49-year-old, gravida 1 parity 1, female presents to a rural emergency room with a painful right groin mass, fever, and chills for the last three days. The patient characterized the pain as sharp and worsened with movement. The patient complained of post-coital vaginal bleeding and a palpable vaginal mass for the last five years. In addition, the patient reported a rash on her torso and lower extremities for the last nine months that was being empirically treated for scabies at the time.

The patient had a past medical history of hypertension, type 2 diabetes mellitus, and hyperlipidemia. Her past surgical history was remarkable for a prior total hysterectomy for benign causes. The patient reports that she tested negative for sexually transmitted infections (STIs) five years ago and had no previous history of STIs. In addition, the patient had not had a pap smear since her hysterectomy, since pap smears are usually discontinued after a total hysterectomy was completed for benign causes.

Upon further workup, a complete blood count (CBC) was remarkable for leukocytosis at 16,800/mm^3 ^(Table 1). Urinalysis revealed 3+ leukocyte esterase, +1 bacteria, and 2+ blood with 11-25 white blood cells (WBCs). Blood cultures, chlamydia, and gonorrhea polymerase chain reaction (PCR), treponema pallidum particle agglutination (TPPA), and rapid plasma regain (RPR) were obtained. A CT scan of the abdomen and pelvis was obtained, which demonstrated an ovoid-shaped mass in the right groin measuring 3.5 x 4.9 cm with surrounding inflammation (Figure 2). The surrounding tissues including the bladder, adnexa, and retroperitoneum were unremarkable.

**Table 1:** Patient's CBC on presentation. RBC: Red blood cells; WBC: white blood cells; MCV: mean corpuscular volume; MCH: mean corpuscular hemoglobin; MCHC: mean corpuscular hemoglobin concentration; MPV: mean platelet volume; RDWCV: red blood cell distribution width - coefficient of variation

Component	Patient’s Value (Units)	Reference Range (Units)
RBC	4.41 x 10^6^/uL	4.2-5.4 x 10^6^/uL
WBC	16.8 x 10^3^/uL	4.5-12.5 10^3^/uL
Hemoglobin	14.4 g/dL	12-16 g/dL
Hematocrit	41.7%	36-48%
MCV	94.5 fL	81-99 fL
MCH	30.6 pg	27-31 pg
MCHC	34.5 g/dL	31-36 g/dL
MPV	10 fL	7.4-10.4 fL
Platelets	138 x 10^3^/uL	150-450 x 10^3^/uL
RDWCV	13.4 %	11.7-14.4%

**Figure 1:**
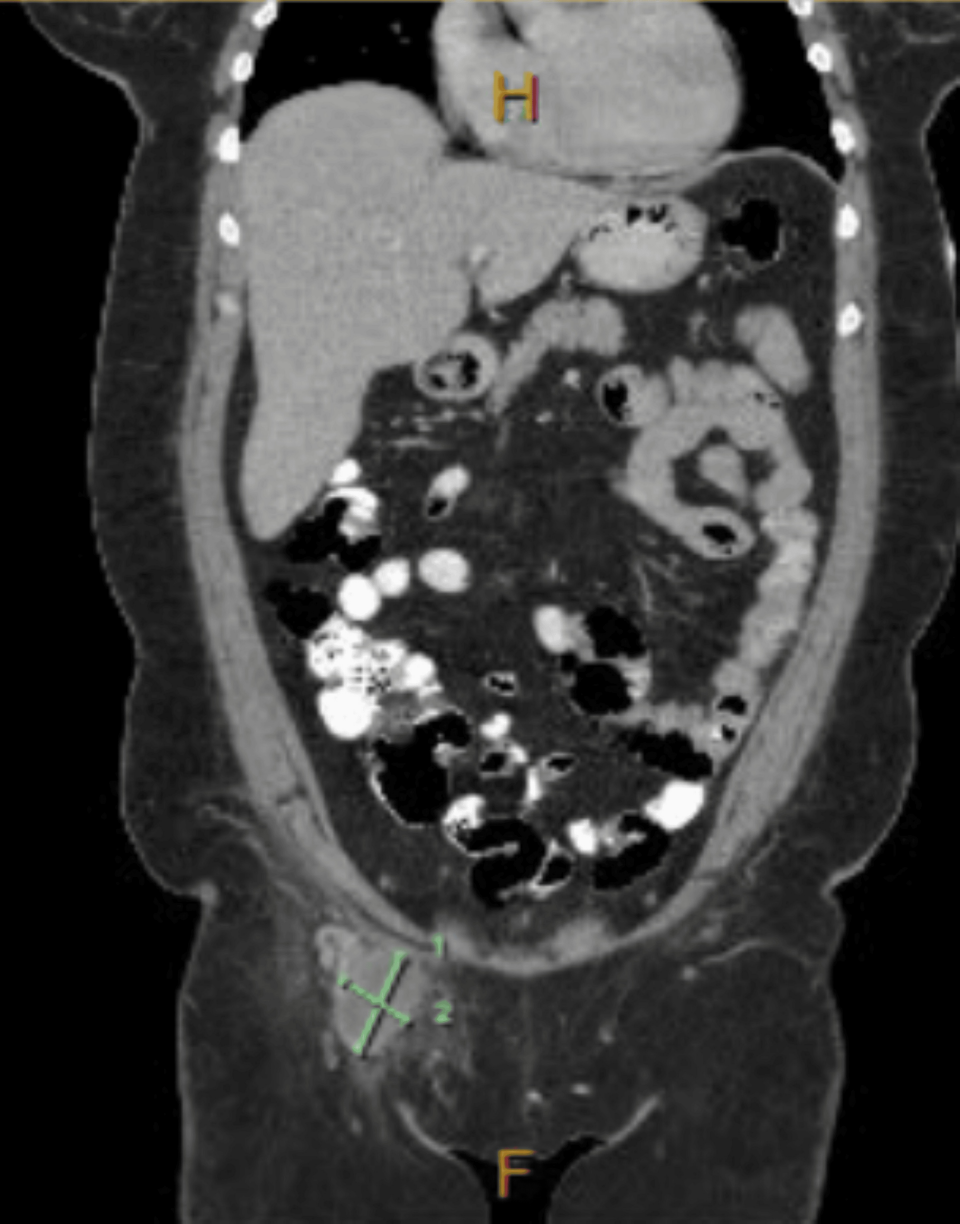
Coronal section of abdomen MRI, the green markers showing the large inguinal lymphadenopathy that measured 3.5 cm x 4.9 cm.

After the results of the CT scan were interpreted, the patient was taken back to the operating room. While under anesthesia, the patient had a vaginal exam done by the gynecology team to evaluate the vaginal mass. During the gynecological examination of the case, an exophytic mass was discovered on the labia majora bilaterally. The left labia majora was entirely infiltrated by the exophytic cauliflower-like lesion and appeared to infiltrate the deeper tissue. The mass measured 3 x 3 cm in the superior and inferior direction and extended 6 cm into the vagina sparing the vaginal cuff. Cystoscopy and rectal exam were performed to determine the extent of the disease and was found to be unremarkable.

Additionally, she underwent a lymphadenectomy performed by the general surgery team. An incision was made on the right inguinal lymphadenopathy and dissected down to the enlarged lymph node. The lymph node was found to be necrotic, prompting the surgery team to incise and drain exudative material. The lymph nodes were dissected, and culture of the exudative material was collected and sent to pathology. The cavity was irrigated, and a drain was placed.

**Figure 2:**
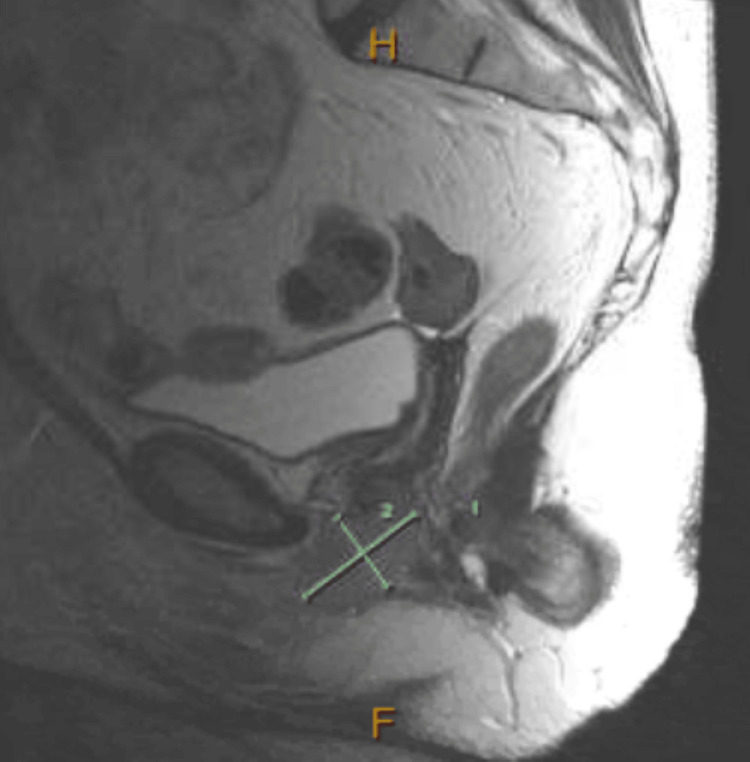
Sagittal section of Pelvic MRI demonstrating the vaginal carcinoma measuring 4.3 cm x 2.7 cm.

**Figure 3:**
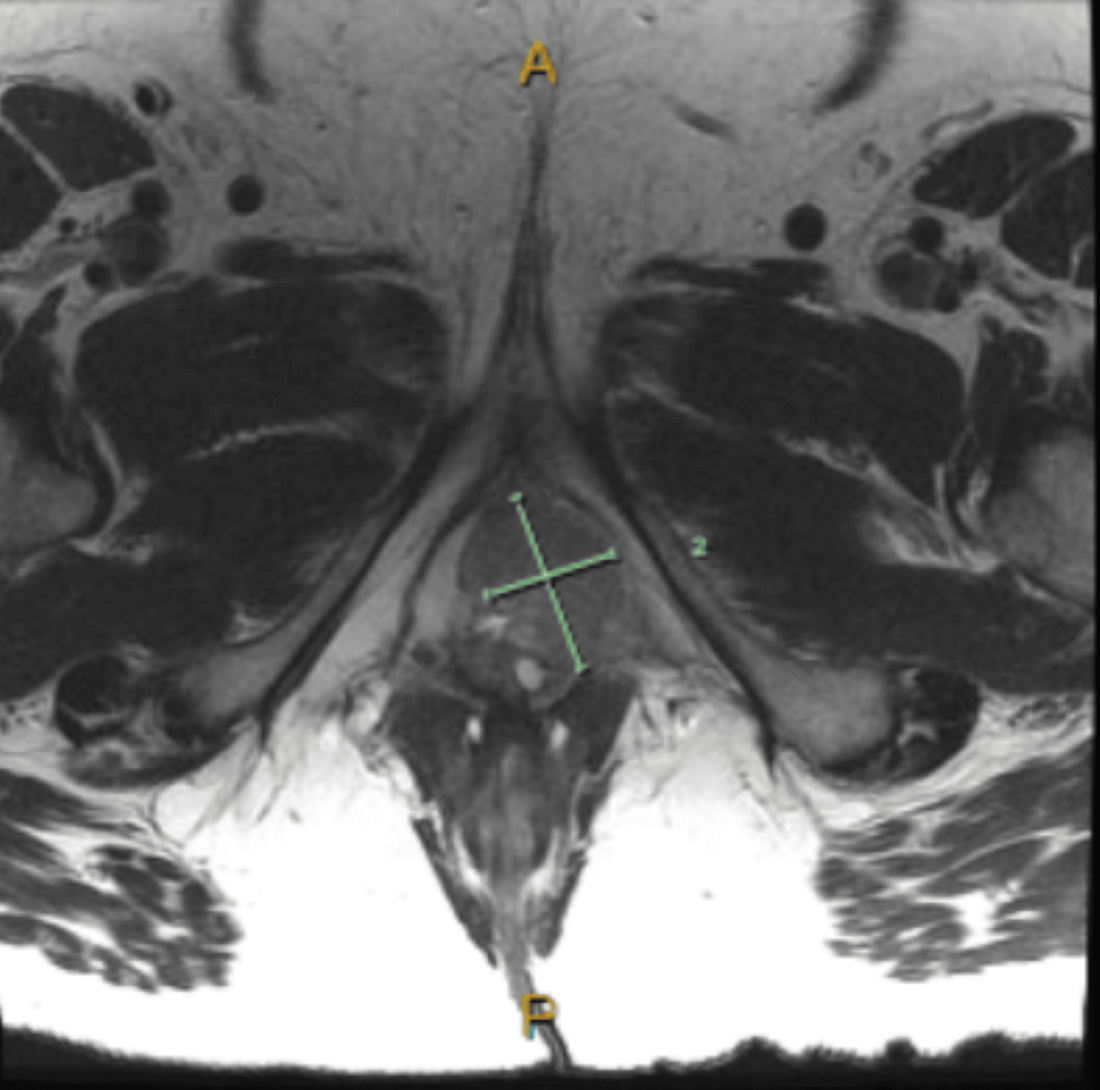
Transverse section of the pelvis MRI demonstrating the malignancy measuring 4.3 cm x 2.7 cm.

The tissue pathology from the lymph node demonstrated metastatic squamous cell carcinoma with tumor necrosis. An anaerobic culture that was collected from the purulent material in the lymph node was found to have a heavy growth of *Fusobacterium necrophorum*. The excisional biopsy that was taken from the vaginal mass was identified as high-grade squamous cell carcinoma. The patient was referred to radiation-oncology and gynecology-oncology for the evaluation of vulvar malignancy. When the patient presented for the consultation with radiation-oncology, the tumor was categorized as stage IIIB (cT1b, cN1b, cM0). Oncology planned treating the malignancy with pelvic radiations with concurrent weekly cisplatin. A follow-up MRI of the pelvis was obtained and demonstrated squamous cell carcinoma involving bilateral labia majora, with the left labia being a more invasive disease than the right side. The malignancy measured 4.3 cm x 2.7 cm. The malignancy invaded the vaginal wall with associated external iliac, inguinal, and left obturator lymphadenopathy (Figures 2, 3).

In addition to the tissue biopsies and cultures, the initial labs that were ordered showed that the patient’s chlamydia and gonorrhea PCR were negative. Additionally, the patient had a negative RPR; however, the TPPA was positive. The patient was diagnosed with latent syphilis. Initially, the patient was treated with amoxicillin and clavulanic acid (Augmentin) for two weeks. Additionally, the patient was given Benzathine penicillin G 7.2 million units total, administered as three doses of 2.4 million units IM (intramuscular) each at one-week intervals to treat late latent syphilis. 

The patient was followed by radiation-oncology for pelvic radiation daily for six weeks and received weekly cisplatin for two months. The patient underwent a follow-up positron emission tomographic (PET) scan that demonstrated a hypermetabolic left inguinal lymph node. Another round of chemotherapy was planned in the following months. 

## Discussion

This case is unique in the fact that vulvar cancer was diagnosed with associated metastatic-infected lymph nodes. The pathogen that was isolated from this patient’s metastatic necrotic lymph nodes was *Fusobacterium necrophorum*. This pathogen is part of the normal flora of the oral and gut compartments and is cited in pharyngitis, tonsillitis, and local abscesses. Tonsillitis can be further complicated with thrombophlebitis within the internal jugular vein, thus resulting in sepsis; this condition is known as Lemierre’s syndrome [7,10,14]. This pathogen has also been identified in gynecology pathologies. *F. necrophorum* has been associated with gynecological infections, including the cases of tubo-ovarian and uterine abscess [11]. This pathogen has demonstrated the pathogenesis to be able to generate infections within different tissues of the human body.

However, what makes this pathogen particularly more clinically relevant is the possibility that *F. necrophorum* may be oncogenic. Regarding malignancies, *F. necrophorum* has been cited in colorectal cancer; however, it has not been described in any gynecological malignancies. A recent study attempting to elucidate the link between *Fusobacteria* and colorectal cancer concluded that this is evidence supporting the notion that an overabundance of *F. necrophorum* may play a role in the pathogenesis of colorectal cancer. This study suggests that the identification of systemic infection via this pathogen should undergo further testing to rule out underlying colorectal cancer [15]. This study described a case in which the pathogen was found within the colorectal tumor cells themselves. More evidence supporting this claim comes from a retrospective study identifying most comorbidities found in cases of *Fusobacterium* infections were underlying cancer or receiving dialysis in older patients [16]. The mechanism of the interaction between the pathogen and cancer is not completely understood at this time. It has been proposed that there is an interaction between the bacteria and the host’s immune system, namely T-cell. The proposed mechanism describes *Fusobacterium*'s ability to inhibit T-cell proliferation and could induce apoptosis of this cell line. The decreasing population of T-cells then could further lead to impaired host response to tumor suppression [17]. Additional proposed mechanisms have been suggested such as an interaction of *Fusobacterium*’s FadA adhesin and E-cadherin/β-catenin molecules, leading to a carcinogenesis pathway [18].

This patient’s past medical history was further complicated by a diagnosis of latent syphilis. Certain STIs have been implicated in the development of invasive vulvar cancer. One study found that patients with invasive vulvar cancer had a history of genital warts, condyloma, gonorrhea, or seropositivity to herpes simplex virus 2 (HSV-2). This study noted that four participants had a diagnosis of syphilis and all four of them had in situ vulvar cancer [19]. These results highlight the importance of the clinician’s role in primary prevention and education. Clinicians should encourage patients to get the HPV vaccine prior to beginning sexual activity. They should also provide education to their patients about safe sexual practices to avoid an STI.

## Conclusions

This case highlights the complexity of vulvar cancer complicated by metastatic lymph nodes infected with *Fusobacterium necrophorum*. The identification of *Fusobacterium* *necrophorum* in this context is notable, given its more common association with conditions like colorectal cancer. This case not only illustrates the diverse clinical manifestations of this pathogen but also emphasizes the critical need for timely and appropriate management, including anaerobic antibiotic coverage, to address both the malignancy and associated infections effectively.

Additionally, the co-diagnosis of latent syphilis in this patient adds another layer of complexity, highlighting the potential interplay between STIs and the development of invasive vulvar cancer. This underscores the clinician’s role in primary prevention, patient education, and the importance of HPV vaccination and safe sexual practices to reduce the risk of such malignancies. The potential link between *Fusobacterium necrophorum* and cancer, while still not fully understood, suggests a need for further research to explore this association and its implications in oncology. This case highlights vulvar cancer with atypical presentations and reinforces the importance of a multidisciplinary approach in treating such complex cases.
